# Supplementary material for: Transcriptome Analysis Reveals Vimentin-Induced Disruption of Cell–Cell Associations Augments Breast Cancer Cell Migration
Source: Cells. 2022 Dec 13;11(24):4035. doi: 10.3390/cells11244035 (PMC9776984; doi:10.3390/cells11244035)
Supplement: Supplementary file 1 [file cells-11-04035-s001.zip › DEGs and enrichment analysis.zip!/DEGs and enrichment analysis/WT_VIMvsVector KEGG/ALL/WT_VIMvsVector_ALL.pathway_map.zip!/WT_VIMvsVector_ALL.pathway_map/src/hsa04071.html]

hsa04071


- hsa:53637

- Up regulated genes

ENSG00000180739(4.10779050911213)

- hsa:123099

- Up regulated genes

ENSG00000168350(2.69521304057358)

- hsa:340485

- Down regulated genes

ENSG00000177076(-2.17137879414265)

- hsa:596

- Up regulated genes

ENSG00000171791(2.03324203294485)

- hsa:624

- Down regulated genes

ENSG00000168398(-1.85119834379304)

Close
